# Supplementary material for: Efficacy of Lajjabati (Mimosa pudica) and Daruchini (Cinnamomum verum) extracts on wound healing in rabbits
Source: PLoS One. 2026 Feb 13;21(2):e0342449. doi: 10.1371/journal.pone.0342449 (PMC12904443; doi:10.1371/journal.pone.0342449)
Supplement: S1 Table — (DOCX) [file pone.0342449.s001.docx]

**S 1 Table.**

**Statistical comparison (p-values and confidence intervals) for the area of swelling and elevation of the wound**

| **ANOVA** | | | | | | |
| --- | --- | --- | --- | --- | --- | --- |
|  | | Sum of Squares | df | Mean Square | F | Sig. |
| swelling | Between Groups | 2.961 | 3 | .987 | 2.656 | .068 |
|  | Within Groups | 10.406 | 28 | .372 |  |  |
|  | Total | 13.367 | 31 |  |  |  |
| elevation | Between Groups | 6.071 | 3 | 2.024 | 15.669 | .000 |
|  | Within Groups | 3.616 | 28 | .129 |  |  |
|  | Total | 9.687 | 31 |  |  |  |

**Post Hoc Tests (Tukey HSD)**

| **Dependent Variable** | | **Group** | **Group** | **Sig.** | **95% Confidence Interval** | |
| --- | --- | --- | --- | --- | --- | --- |
|  |  |  |  |  | **Lower Bound** | **Upper Bound** |
| swelling |  | Group A | Group B | .926 | -.6447 | 1.0197 |
|  |  |  | Group C | .373 | -1.3322 | .3322 |
|  |  |  | Group D | .373 | -1.3322 | .3322 |
|  |  | Group B | Group A | .926 | -1.0197 | .6447 |
|  |  |  | Group C | .133 | -1.5197 | .1447 |
|  |  |  | Group D | .133 | -1.5197 | .1447 |
|  |  | Group C | Group A | .373 | -.3322 | 1.3322 |
|  |  |  | Group B | .133 | -.1447 | 1.5197 |
|  |  |  | Group D | 1.000 | -.8322 | .8322 |
|  |  | Group D | Group A | .373 | -.3322 | 1.3322 |
|  |  |  | Group B | .133 | -.1447 | 1.5197 |
|  |  |  | Group C | 1.000 | -.8322 | .8322 |
| elevation |  | Group A | Group B | .000 | .4719 | 1.4531 |
|  |  |  | Group C | .985 | -.5531 | .4281 |
|  |  |  | Group D | .985 | -.5531 | .4281 |
|  |  | Group B | Group A | .000 | -1.4531 | -.4719 |
|  |  |  | Group C | .000 | -1.5156 | -.5344 |
|  |  |  | Group D | .000 | -1.5156 | -.5344 |
|  |  | Group C | Group A | .985 | -.4281 | .5531 |
|  |  |  | Group B | .000 | .5344 | 1.5156 |
|  |  |  | Group D | 1.000 | -.4906 | .4906 |
|  |  | Group D | Group A | .985 | -.4281 | .5531 |
|  |  |  | Group B | .000 | .5344 | 1.5156 |
|  |  |  | Group C | 1.000 | -.4906 | .4906 |
