# Supplementary material for: Efficacy of Lajjabati (Mimosa pudica) and Daruchini (Cinnamomum verum) extracts on wound healing in rabbits
Source: PLoS One. 2026 Feb 13;21(2):e0342449. doi: 10.1371/journal.pone.0342449 (PMC12904443; doi:10.1371/journal.pone.0342449)
Supplement: S2 Table — (DOCX) [file pone.0342449.s002.docx]

**S2 Table.**

**Statistical comparison (p-values and confidence intervals) for the Width (mm) of the sutured area of wounds at Day-0 to day-21 of different groups**

| **ANOVA** | | | | | | |
| --- | --- | --- | --- | --- | --- | --- |
|  | | Sum of Squares | df | Mean Square | F | Sig. |
| D0 | Between Groups | 6.773 | 3 | 2.258 | 14.985 | .000 |
|  | Within Groups | 4.219 | 28 | .151 |  |  |
|  | Total | 10.992 | 31 |  |  |  |
| D3 | Between Groups | 5.711 | 3 | 1.904 | 6.248 | .002 |
|  | Within Groups | 8.531 | 28 | .305 |  |  |
|  | Total | 14.242 | 31 |  |  |  |
| D7 | Between Groups | 2.836 | 3 | .945 | 2.134 | .118 |
|  | Within Groups | 12.406 | 28 | .443 |  |  |
|  | Total | 15.242 | 31 |  |  |  |
| D14 | Between Groups | 5.125 | 3 | 1.708 | 12.756 | .000 |
|  | Within Groups | 3.750 | 28 | .134 |  |  |
|  | Total | 8.875 | 31 |  |  |  |
| D21 | Between Groups | .961 | 3 | .320 | 1.876 | .157 |
|  | Within Groups | 4.781 | 28 | .171 |  |  |
|  | Total | 5.742 | 31 |  |  |  |

**Post Hoc Tests (Tukey HSD)**

| Dependent Variable | | Group | Group | Sig. | 95% Confidence Interval | |
| --- | --- | --- | --- | --- | --- | --- |
|  |  |  |  |  | Lower Bound | Upper Bound |
| D0 |  | Group A | Group B | .000 | .5326 | 1.5924 |
|  |  |  | Group C | 1.000 | -.5299 | .5299 |
|  |  |  | Group D | 1.000 | -.5299 | .5299 |
|  |  | Group B | Group A | .000 | -1.5924 | -.5326 |
|  |  |  | Group C | .000 | -1.5924 | -.5326 |
|  |  |  | Group D | .000 | -1.5924 | -.5326 |
|  |  | Group C | Group A | 1.000 | -.5299 | .5299 |
|  |  |  | Group B | .000 | .5326 | 1.5924 |
|  |  |  | Group D | 1.000 | -.5299 | .5299 |
|  |  | Group D | Group A | 1.000 | -.5299 | .5299 |
|  |  |  | Group B | .000 | .5326 | 1.5924 |
|  |  |  | Group C | 1.000 | -.5299 | .5299 |
| D3 |  | Group A | Group B | .051 | -.0035 | 1.5035 |
|  |  |  | Group C | .802 | -1.0035 | .5035 |
|  |  |  | Group D | .673 | -1.0660 | .4410 |
|  |  | Group B | Group A | .051 | -1.5035 | .0035 |
|  |  |  | Group C | .006 | -1.7535 | -.2465 |
|  |  |  | Group D | .003 | -1.8160 | -.3090 |
|  |  | Group C | Group A | .802 | -.5035 | 1.0035 |
|  |  |  | Group B | .006 | .2465 | 1.7535 |
|  |  |  | Group D | .996 | -.8160 | .6910 |
|  |  | Group D | Group A | .673 | -.4410 | 1.0660 |
|  |  |  | Group B | .003 | .3090 | 1.8160 |
|  |  |  | Group C | .996 | -.6910 | .8160 |
| D7 |  | Group A | Group B | .189 | -.2212 | 1.5962 |
|  |  |  | Group C | 1.000 | -.9087 | .9087 |
|  |  |  | Group D | 1.000 | -.9087 | .9087 |
|  |  | Group B | Group A | .189 | -1.5962 | .2212 |
|  |  |  | Group C | .189 | -1.5962 | .2212 |
|  |  |  | Group D | .189 | -1.5962 | .2212 |
|  |  | Group C | Group A | 1.000 | -.9087 | .9087 |
|  |  |  | Group B | .189 | -.2212 | 1.5962 |
|  |  |  | Group D | 1.000 | -.9087 | .9087 |
|  |  | Group D | Group A | 1.000 | -.9087 | .9087 |
|  |  |  | Group B | .189 | -.2212 | 1.5962 |
|  |  |  | Group C | 1.000 | -.9087 | .9087 |
| D14 |  | Group A | Group B | .000 | .3754 | 1.3746 |
|  |  |  | Group C | .903 | -.6246 | .3746 |
|  |  |  | Group D | 1.000 | -.4996 | .4996 |
|  |  | Group B | Group A | .000 | -1.3746 | -.3754 |
|  |  |  | Group C | .000 | -1.4996 | -.5004 |
|  |  |  | Group D | .000 | -1.3746 | -.3754 |
|  |  | Group C | Group A | .903 | -.3746 | .6246 |
|  |  |  | Group B | .000 | .5004 | 1.4996 |
|  |  |  | Group D | .903 | -.3746 | .6246 |
|  |  | Group D | Group A | 1.000 | -.4996 | .4996 |
|  |  |  | Group B | .000 | .3754 | 1.3746 |
|  |  |  | Group C | .903 | -.6246 | .3746 |
| D21 |  | Group A | Group B | .288 | -.1891 | .9391 |
|  |  |  | Group C | .990 | -.6266 | .5016 |
|  |  |  | Group D | 1.000 | -.5641 | .5641 |
|  |  | Group B | Group A | .288 | -.9391 | .1891 |
|  |  |  | Group C | .172 | -1.0016 | .1266 |
|  |  |  | Group D | .288 | -.9391 | .1891 |
|  |  | Group C | Group A | .990 | -.5016 | .6266 |
|  |  |  | Group B | .172 | -.1266 | 1.0016 |
|  |  |  | Group D | .990 | -.5016 | .6266 |
|  |  | Group D | Group A | 1.000 | -.5641 | .5641 |
|  |  |  | Group B | .288 | -.1891 | .9391 |
|  |  |  | Group C | .990 | -.6266 | .5016 |
