# Supplementary material for: Efficacy of Lajjabati (Mimosa pudica) and Daruchini (Cinnamomum verum) extracts on wound healing in rabbits
Source: PLoS One. 2026 Feb 13;21(2):e0342449. doi: 10.1371/journal.pone.0342449 (PMC12904443; doi:10.1371/journal.pone.0342449)
Supplement: S1 Appendix — (DOCX) [file pone.0342449.s003.docx]

**S1 Appendix.**

**Statistical comparison of bacterial colony count with the statistical differences among different groups (Mean, SE and 95% confidence interval)**

| Groups | Mean | Std. Error | 95% Confidence Interval for Mean | |
| --- | --- | --- | --- | --- |
|  |  |  | Lower Bound | Upper Bound |
| G-A | 10.13 | 1.231 | 7.21 | 13.04 |
| G-B | 25.63 | 4.088 | 15.96 | 35.29 |
| G-C | 31.00 | 3.891 | 21.80 | 40.20 |
| G-D | 35.38 | 3.741 | 26.53 | 44.22 |
| Total | 25.53 | 2.369 | 20.70 | 30.36 |

| **ANOVA** | | | | | |
| --- | --- | --- | --- | --- | --- |
|  | Sum of Squares | df | Mean Square | F | Sig. |
| Between Groups | 2913.344 | 3 | 971.115 | 10.251 | .000 |
| Within Groups | 2652.625 | 28 | 94.737 |  |  |
| Total | 5565.969 | 31 |  |  |  |

**Post Hoc Tests**

| Group | (J) Group | Sig. | 95% Confidence Interval | |
| --- | --- | --- | --- | --- |
|  |  |  | Lower Bound | Upper Bound |
| G-A | G-B | .018 | -28.79 | -2.21 |
|  | G-C | .001 | -34.16 | -7.59 |
|  | G-D | .000 | -38.54 | -11.96 |
| G-B | G-A | .018 | 2.21 | 28.79 |
|  | G-C | .690 | -18.66 | 7.91 |
|  | G-D | .211 | -23.04 | 3.54 |
| G-C | G-A | .001 | 7.59 | 34.16 |
|  | G-B | .690 | -7.91 | 18.66 |
|  | G-D | .805 | -17.66 | 8.91 |
| G-D | G-A | .000 | 11.96 | 38.54 |
|  | G-B | .211 | -3.54 | 23.04 |
|  | G-C | .805 | -8.91 | 17.66 |
