# Supplementary material for: Efficacy of Lajjabati (Mimosa pudica) and Daruchini (Cinnamomum verum) extracts on wound healing in rabbits
Source: PLoS One. 2026 Feb 13;21(2):e0342449. doi: 10.1371/journal.pone.0342449 (PMC12904443; doi:10.1371/journal.pone.0342449)
Supplement: S1 File — (DOCX) [file pone.0342449.s004.docx]

**Dataset**

**Dataset for Figure 2**

**Data of Bacterial Colony Count (per 100 microlitre) per day**

| **Group** | **Day** | **Bacterial Count per 100 microlitre** |
| --- | --- | --- |
| G-A | D-1 | 15 |
| G-A | D-2 | 14 |
| G-A | D-3 | 12 |
| G-A | D-4 | 11 |
| G-A | D-5 | 9 |
| G-A | D-6 | 8 |
| G-A | D-7 | 7 |
| G-A | D-8 | 5 |
| G-B | D-1 | 41 |
| G-B | D-2 | 37 |
| G-B | D-3 | 34 |
| G-B | D-4 | 29 |
| G-B | D-5 | 24 |
| G-B | D-6 | 17 |
| G-B | D-7 | 14 |
| G-B | D-8 | 9 |
| G-C | D-1 | 48 |
| G-C | D-2 | 45 |
| G-C | D-3 | 36 |
| G-C | D-4 | 29 |
| G-C | D-5 | 27 |
| G-C | D-6 | 24 |
| G-C | D-7 | 21 |
| G-C | D-8 | 18 |
| G-D | D-1 | 49 |
| G-D | D-2 | 46 |
| G-D | D-3 | 41 |
| G-D | D-4 | 39 |
| G-D | D-5 | 35 |
| G-D | D-6 | 31 |
| G-D | D-7 | 23 |
| G-D | D-8 | 19 |

**Dataset for Table 1**

**The effects of Lajjabati paste (Group A), Daruchini paste (Group B), their mixture (Group C), and normal saline (Group D) on wound healing in rabbits**

| Groups | Area of Swelling of Wound (mm)  D0-D3 | Elevation of Wound (mm)  D0-D7 |
| --- | --- | --- |
| G-A | 11 | 3 |
| G-A | 11.5 | 3.5 |
| G-A | 12.5 | 4 |
| G-A | 12 | 3.5 |
| G-A | 12.5 | 4 |
| G-A | 11.5 | 3.5 |
| G-A | 11 | 3 |
| G-A | 11.5 | 3.5 |
| G-B | 12 | 2.6 |
| G-B | 11.5 | 2.5 |
| G-B | 12 | 3 |
| G-B | 11.5 | 2 |
| G-B | 11 | 2.4 |
| G-B | 11.5 | 2.5 |
| G-B | 11.5 | 2.5 |
| G-B | 11 | 2.8 |
| G-C | 11 | 3 |
| G-C | 12 | 3.5 |
| G-C | 12.5 | 4 |
| G-C | 11.5 | 3.3 |
| G-C | 13 | 3.7 |
| G-C | 12.5 | 3.5 |
| G-C | 12 | 4 |
| G-C | 13 | 3.5 |
| G-D | 11 | 3 |
| G-D | 12 | 3.5 |
| G-D | 12.5 | 4 |
| G-D | 11.5 | 3.5 |
| G-D | 13 | 4 |
| G-D | 12.5 | 3.5 |
| G-D | 12 | 4 |
| G-D | 13 | 3 |

**Dataset for Table 2**

**Width (mm) of the sutured area of wounds treated with lajjabati (Group A), daruchini (Group B), a mixture of lajjabati and daruchini (Group C), and normal saline (Group D) on wound healing in rabbits**

| Group | D0 | D3 | D7 | D14 | D21 |
| --- | --- | --- | --- | --- | --- |
| G-A | 7.5 | 7.5 | 7 | 6.5 | 5.5 |
| G-A | 7 | 7.5 | 7 | 7 | 5 |
| G-A | 7.5 | 8 | 7.5 | 7 | 5.5 |
| G-A | 8 | 8.5 | 8 | 7.5 | 5 |
| G-A | 7.5 | 8 | 8 | 7 | 4.5 |
| G-A | 8 | 9 | 8.5 | 7.5 | 5.5 |
| G-A | 7.5 | 8.5 | 8 | 7 | 5 |
| G-A | 7 | 7.5 | 7.5 | 6.5 | 4.5 |
| G-B | 6 | 7 | 7 | 6 | 4 |
| G-B | 7 | 8 | 7 | 6 | 4.5 |
| G-B | 7 | 7.5 | 7.5 | 6.5 | 5.5 |
| G-B | 6.5 | 7.5 | 8 | 6.5 | 5 |
| G-B | 6 | 7 | 6.5 | 6 | 4.5 |
| G-B | 6.5 | 7 | 6.5 | 5.5 | 4.5 |
| G-B | 6 | 7.5 | 7 | 6.5 | 5 |
| G-B | 6.5 | 7 | 6.5 | 6 | 4.5 |
| G-C | 7 | 7.5 | 6.5 | 7 | 5.5 |
| G-C | 7.5 | 8.5 | 7 | 6.5 | 5 |
| G-C | 7 | 8 | 7 | 7 | 5.5 |
| G-C | 8 | 8.5 | 8 | 7.5 | 5 |
| G-C | 7.5 | 8 | 8 | 7 | 4.5 |
| G-C | 8 | 9.5 | 9 | 7.5 | 5.5 |
| G-C | 7.5 | 8.5 | 8 | 7 | 5 |
| G-C | 7.5 | 8 | 8 | 7.5 | 5 |
| G-D | 7 | 7.5 | 6.5 | 7 | 5.5 |
| G-D | 7.5 | 8 | 7 | 6.5 | 5 |
| G-D | 7.5 | 8.5 | 7.5 | 7 | 5.5 |
| G-D | 8 | 9 | 8 | 7.5 | 5 |
| G-D | 7.5 | 8.5 | 8 | 7 | 4.5 |
| G-D | 8 | 9.5 | 9 | 7.5 | 5.5 |
| G-D | 7 | 8 | 7.5 | 6.5 | 5 |
| G-D | 7.5 | 8 | 8 | 7 | 4.5 |
