# Supplementary material for: Efficacy of Lajjabati (Mimosa pudica) and Daruchini (Cinnamomum verum) extracts on wound healing in rabbits
Source: PLoS One. 2026 Feb 13;21(2):e0342449. doi: 10.1371/journal.pone.0342449 (PMC12904443; doi:10.1371/journal.pone.0342449)
Supplement: Attachment — Submitted filename: Response to Reviewers.docx [file pone.0342449.s006.docx]

Dear Editor and Reviewer,

We would like to thank the reviewer for the constructive and thoughtful feedback on our manuscript. We have carefully addressed each concern to improve the scientific rigor and clarity of the study. Please find our detailed answers to the reviewer’s comments and suggestions below. Please note the highlighted part in the manuscript for correction.

Kind regards

Rukhsana Amin Runa

**Review Comments to the Author**

*Reviewer #2:*

This manuscript presents interesting preliminary findings; however, several major methodological clarifications and revisions are required. Issues include insufficient detail on randomization and blinding, lack of standardized histological scoring, absence of functional wound-healing measures, inconsistencies in reported results, and overinterpretation of findings beyond the acute rabbit model. With appropriate revision and clarification, the paper could make a meaningful contribution. Therefore, I recommend Major Revision.

*Authors response:*

Below are our point-by-point responses:

**1. Comment: Insufficient detail on randomization and blinding.**

- ***Response:*** We agree that these details are vital for transparency. We have updated the Materials and Methods section (lines 118-120) to explicitly state that rabbits were assigned to groups using a random lottery system. Additionally, we have clarified that the histopathological analysis was performed by investigators blinded to the treatment groups to prevent observational bias.

**2. Comment: Lack of standardized histological scoring.**

- ***Response:*** We have added the descriptive observations with a semi-quantitative scoring system (0–3 scale) for inflammation, fibrosis, and glandular changes in the materials and methods section (lines 181-184). This new data has also been added as Table 4 in the results section (lines 238-245).

**3. Comment: Absence of functional wound-healing measures.**

- ***Response:*** We acknowledge that biomechanical data (such as tensile strength) would strengthen the study. Since this was a pilot study, we focused on morphological and histo-architectural changes. We have now included this as a specific limitation in the Discussion section and highlighted it as a priority for our follow-up research (lines 352-354; 375-377).

**4. Comment: Inconsistencies in reported results.**

- ***Response:*** We have clarified this in the result (lines 199-201; 206-207) and discussion section (lines 311-316)

**5. Comment: Overinterpretation of findings beyond the acute rabbit model.**

- ***Response:*** We have toned down the conclusions throughout the manuscript. We have also added a section on limitations regarding the sample size (n=4) and the need for broader clinical trials (lines 365-377).
